# Detailed Structural Characterization of Oxidized Sucrose and Its Application in the Fully Carbohydrate-Based Preparation of a Hydrogel from Carboxymethyl Chitosan

**DOI:** 10.3390/molecules27186137

**Published:** 2022-09-19

**Authors:** Hiroyuki Kono, Junki Noda, Haruki Wakamori

**Affiliations:** 1 Division of Applied Chemistry and Biochemistry, National Institute of Technology, Tomakomai College, Nishikioka 443, Tomakomai 059 1275, Japan; 2 Hokkaido Soda Co. Ltd., Numanohata 134-122, Tomakomai 059 1364, Japan

**Keywords:** oxidized sucrose, polysaccharide hydrogel, carboxymethyl chitosan, cross-linking agent, NMR

## Abstract

Oxidized sucrose (OS) is a bio-based cross-linking agent with excellent biological safety and environmental non-toxicity. However, the precise structure of OS has not been elucidated owing to its structural complexity and low purity. Accordingly, in this study, complete chemical shift assignments were performed by applying various nuclear magnetic resonance techniques, which permitted the structural and quantitative characterization of the two main OS products, each of which contained four aldehyde groups. In addition, we investigated the use of OS as a cross-linking agent in the preparation of a hydrogel from carboxymethyl chitosan (CMC), one of the most popular polysaccharides for use in biomedical applications. The primary amine groups of CMC were immediately cross-linked with the aldehyde groups of OS to form hydrogels without the requirement for a catalyst. It was found that the degree of cross-linking could be easily controlled by the feed amount of OS during CMC hydrogel preparation and the final cross-linking degree affected the thermal, swelling, and rheological properties of the obtained hydrogel. The results presented in this study are therefore expected to be applicable in the preparation of fully carbohydrate-based hydrogels for medical and pharmaceutical applications.

## 1. Introduction

Hydrogels are three-dimensional networks of hydrophilic polymers in which individual polymer chains are chemically or physically cross-linked, allowing them to absorb water or saline solutions, and retain large volumes of these solutions while maintaining their structures [1]. In addition, hydrogels possess a flexibility similar to that of natural tissues because of their high-water content, and as a result, a variety of synthetic or natural hydrogels were widely used in biomedical and pharmaceutical processes, including drug and gene delivery, tissue engineering, wound healing, and cosmetic manufacturing [2,3]. Among the various matrices employed for hydrogel preparation, chitosan and chitosan derivatives are commonly used because of their unique properties, such as their non-toxicity, biocompatibility, biodegradability, bioactivity, antimicrobial activity, and mucoadhesive properties [4,5].

Chitosan is obtained by the deacetylation of chitin, which comprises linear *β*-(1→4)-*N*-acetyl glucosamine (GlcNAc) residues. As a result, chitosan is composed of linear *β*-(1→4)-glucosamine (GlcN) residues and little or no GlcNAc, wherein the molar percentage of GlcN units for all residues (i.e., the deacetylation degree) depends on the deacetylation of chitin. Chitosan is soluble in acidic solutions, forming a polycationic polymer with a high density of positive ammonium groups; however, it is not soluble at neutral and physiological pH because of its stable crystalline structure. This issue of water insolubility can be overcome by preparing water-soluble derivatives. More specifically, among the known water-soluble chitosan derivatives, carboxymethyl chitosan (CMC) is widely studied because of its ease of synthesis and potential applications, including wound healing, bio-imaging, tissue engineering, and drug/gene delivery [6,7]. CMC and chitosan are degraded by enzymes in the body, such as lysozyme and chitosanase, to produce oligosaccharides and monosaccharides, which are subsequently absorbed by the body [8]. Thus, a cross-linking agent is required to prepare chemical hydrogels from chitosan and/or chitosan derivatives. In addition, to prepare hydrogels for medical and pharmaceutical applications, it is also necessary to select non-toxic and biocompatible reaction solvents and catalysts. The most common cross-linking agents for the preparation of hydrogels from chitosan and its derivatives are glutaraldehyde, formaldehyde, epichlorohydrin, and ethylene glycol diglycidyl ether; however, these compounds are extremely toxic and so they are not suitable for biological applications [9,10,11]. In contrast, genipin [12,13], which is a chemical compound found in *Genipa americana* fruit extract, is a natural cross-linking agent that can be used to prepare chitosan hydrogels. However, the high costs associated with this compound render it economically unfeasible for use in this context. Thus, for the development and widespread use of hydrogel materials that utilize the excellent biocompatibility of chitosan and its derivatives, it is necessary to construct a non-toxic, biocompatible, and inexpensive cross-linking agent.

As previously reported, non-toxic oxidized poly- and oligosaccharides are commonly used for cross-linking in hydrogel systems [14,15,16,17,18]. These saccharides are oxidized by periodates under acidic conditions to produce polar or polymeric aldehydes, which are relatively non-toxic and highly reactive. As an example system, the oxidization of cellulose by sodium periodate cleaves the C2–C3 bond of glucose to produce 2,3-dialdehyde cellulose [14]. In addition, the oxidized products of polysaccharides, including alginic acid [14,15], dextran [16], hyaluronic acid [17], and pectin [18], were reported to cross-link biomaterials, such as proteins and polysaccharides. Although polysaccharide aldehydes are abundant in the constituent residues, relatively few aldehyde groups are involved in the cross-linking reactions because of their high molecular weights and bulky backbone structures. Ultimately, this often results in an insufficient cross-linking efficiency when polysaccharide aldehydes are employed. In contrast, oxidized sucrose (OS) can contain up to four aldehyde moieties in its structure, and its high water solubility and relatively small molecular weight allow it to be treated in a similar manner to existing cross-linking agents [19,20]. OS was reported to efficiently cross-link polysaccharides, such as starch [21], schizophyllan [22], and chitosan [23], as well as proteins, such as collagen [24], gelatin [25], and zein from corn protein [19], to enhance the physical and chemical properties of these biomaterials. The biocompatibility of OS was also demonstrated by in vitro cell culture studies of protein scaffolds cross-linked with OS [19,26]. Furthermore, because OS has a polar backbone structure that contains hydroxyl groups and glycosidic bonds, it is not volatile, and so does not exhibit the same environmental toxicity associated with formaldehyde and glutaraldehyde [27]. Based on these factors, OS is expected to act as a bio-based cross-linking agent with an excellent biological safety profile and environmental non-toxicity.

Although the application of OS has resulted in the preparation of biomaterials with enhanced properties, the molecular structure of OS has yet to be analyzed in detail. OS is formed by the action of iodine on sucrose, resulting in C3–C4 cleavage of the fructose residue and C2–C3 cleavage of the glucose residue. Cleavage of the C3–C4 bond of the glucose residue is also considered to take place, suggesting that OS is a mixture of multiple oxidative cleavages [28]. Previous reports [19,20,21,22,23,24,25] have quoted the amount of aldehydes at ~20–45% when full oxidation is reached; however, the precise oxidative cleavage sites of OS have not been confirmed. In addition, although the preparation of hydrogels from polysaccharides was reported using OS, the majority of studies have focused on the physical and chemical properties of the obtained gels, lacking information regarding the correlation between the gel structures and their properties, such as the cross-linking density.

**Figure 1:**
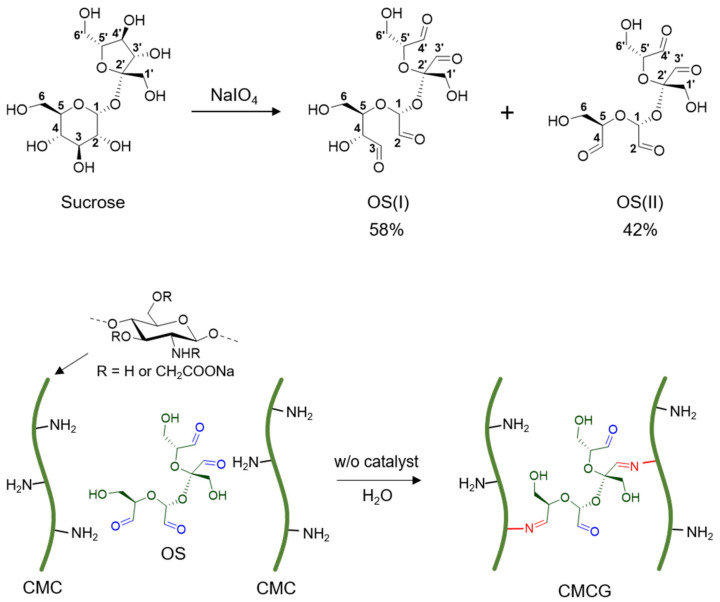
Schematic outlines of the preparation of oxidized sucrose (OS) from sucrose using sodium periodate (NaIO_4_) (top) and the carboxymethyl chitosan hydrogels (CMCG) from carboxymethyl chitosan (CMC) using oxidized sucrose (OS) as a cross-linking agent (bottom).

Therefore, for the purpose of this study, two objectives were set, namely the determination of the molecular structure of OS and the preparation of a CMC hydrogel (CMCG) taking advantage of the cross-linking properties of OS (Figure 1). More specifically, we aim to achieve a complete chemical shift assignment for OS and determine its precise molecular structure, which will be useful for tracing the reaction and elucidating the structure–function relationship. Furthermore, we aim to experimentally clarify the relationship between the various properties of CMCG cross-linked with OS and determine the degree of cross-linking, which will provide useful information for the development of hydrogel materials for medical and pharmaceutical applications.

## 2. Results and Discussion

### 2.1. Structural Characterization of OS

The mechanism of sucrose oxidation was reported previously and the expected structure of the product is a hemiacetal containing four aldehyde groups (OS(II), Figure 1), in which the C2–C3–C4 linkages of the glucose residue and the C3–C4 linkage of the fructose residue are completely oxidized [29]. However, the OS structures reported to date are mixtures of isomeric acetals, giving complex resonance lines in their NMR spectra. This makes it difficult to determine the precise structures and composition rates of OS. Thus, we initially aimed to precisely determine the molecular structure of OS obtained by the oxidation of sucrose.

More specifically, following the oxidation of an aqueous solution of sucrose and sodium periodate, barium chloride was added to remove iodine as a barium iodide precipitate. The aqueous solution was then treated with a cation and anion exchange resin to completely remove the ionic substances and yield OS as a white powder.

The Fourier transform infrared (FTIR) spectrum of sucrose (Figure S1) exhibits absorption peaks between 3500 and 3200 cm^−1^ and between 2950 and 2800 cm^−1^, corresponding to the O–H stretching vibrations and the saturated C–H stretching vibrations, respectively. In addition, the absorptions at 1150 and 960 cm^−1^ correspond to the C–O and C–O–C stretching vibrations, respectively. The FTIR spectrum of OS also exhibited an additional absorption between 1710 and 1690 cm^−1^, which was assigned to the stretching vibrations of the aldehyde C=O groups [28], which were created as a result of the oxidization of sucrose.

**Figure 2:**
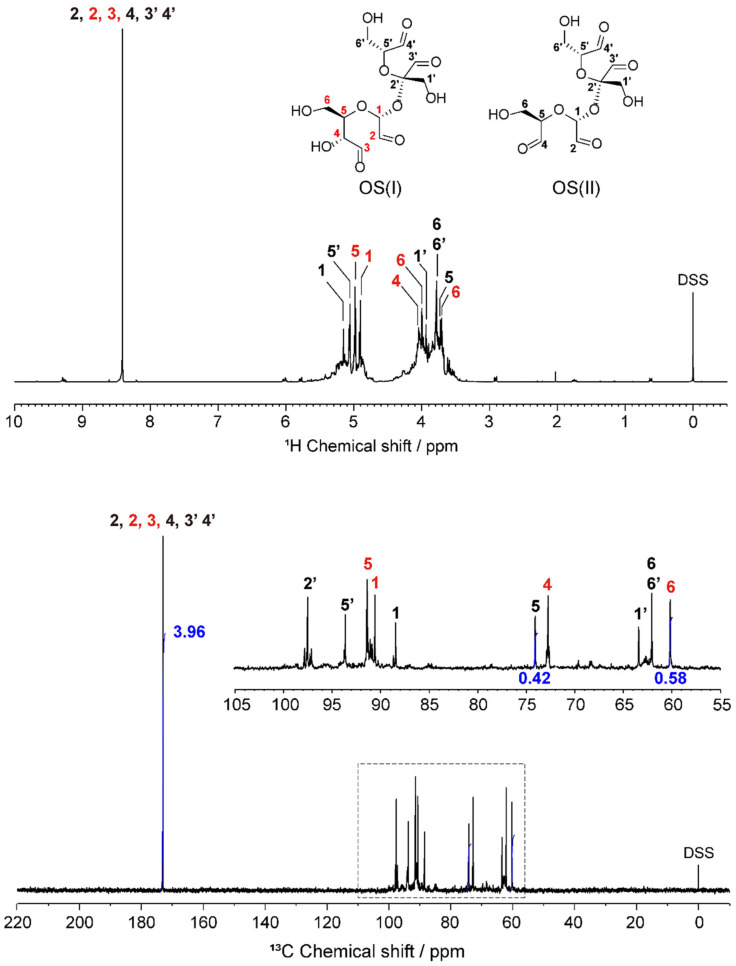
Water-suppressed ^1^H (top) and quantitative ^13^C (bottom) NMR spectra of the oxidized sucrose (OS) in D_2_O at 298 K. Sodium 3-(trimethylsilyl)propane-1-sulfonate (DSS) was used as an internal standard.

Figure 2 shows the water-suppressed ^1^H and quantitative ^13^C NMR spectra of OS dissolved in deuterium oxide (D_2_O). More specifically, the observation of a free aldehyde group through ^1^H and ^13^C signals at 8.4 and 173 ppm confirmed that the oxidation of sucrose generated polyaldehyde derivatives. To precisely determine the structure of OS, complete resonance assignment of these spectra was performed using ^13^C distortionless enhancement by polarization transfer (DEPT) 135, ^1^H–^13^C heteronuclear single quantum coherence (HSQC), and HSQC-total correlation spectroscopy (TOCSY).

Figure S2 shows an expansion (110–55 ppm) of the normal ^13^C and ^13^C DEPT135 spectra of the prepared OS. In the ^13^C DEPT spectrum, primary and tertiary carbon atoms appeared as positive phases, secondary carbon atoms appeared as negative lines, and quaternary carbon atoms were not detected [30]. Based on the ^13^C DEPT135 spectrum of OS, the ^13^C resonances at 98, 95–72, and 65–59 ppm were assigned to the quaternary, tertiary, and secondary carbon atoms, respectively. The quaternary carbon atom in the expected OS structure is the carbon atom at position 2 of the oxidized fructose residue, which corresponds with the resonance at 98 ppm.

**Figure 3:**
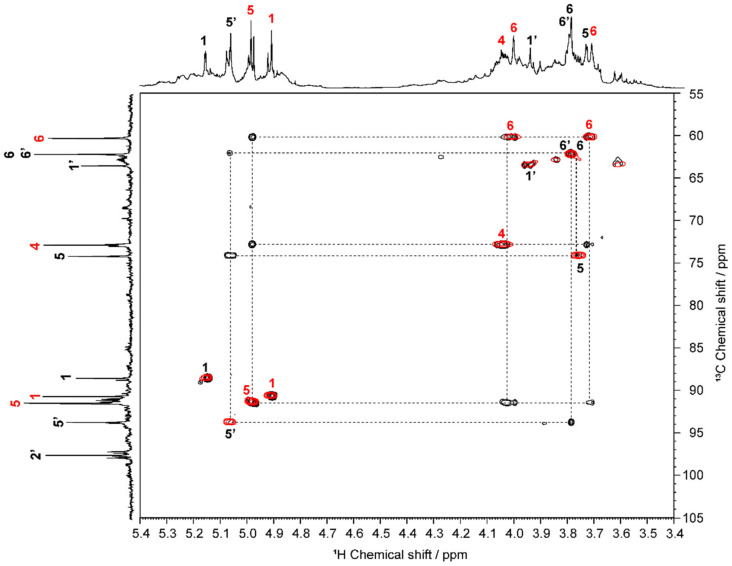
Two-dimensional contour plots of the ^1^H–^13^C heteronuclear single quantum coherence (HSQC), and HSQC-total correlation spectroscopy (TOCSY) spectra of OS (298 K, D_2_O).

Figure 3 shows contour plots of the HSQC and HSQC–TOCSY spectra of the prepared OS. In the HSQC spectrum, correlations between directly coupled ^1^H–^13^C spin pair(s) were observed, whereas in the HSQC–TOCSY spectrum, correlations between long-range ^1^H–^13^C spin couplings and directly coupled spin pair(s) were detected [31,32,33], stacked on the HSQC spectrum. The HSQC–TOCSY spectrum permitted clarification of the correlation between the 5 and 6 positions in the oxidized fructose residue (black numbers 5′ and 6′ in Figure 2). For the oxidized glucose unit, two types of correlation were observed in the spectra, namely a correlation between positions 5 and 6 (black numbers), and a correlation between positions 4, 5, and 6 (red numbers). This finding indicates that the former is a glucose unit in which both the C2–C3 and C3–C4 bonds are oxidatively cleaved, whereas the latter could be attributed to a glucose residue in which only the C2–C3 bonds are oxidatively cleaved. Based on the directly coupled ^1^H–^13^C spin correlations in the HSQC spectrum and the series comparison of each carbon signal in the ^13^C DEPT135 spectrum, the ^1^H and ^13^C chemical shifts of the two main products of OS, namely OS(I) and OS(II), as shown in Figure 1 and Figure 2, could be completely assigned. The full chemical shift data are presented in Table 1. It should also be noted that resonance lines of other species were also observed in the ^1^H and ^13^C NMR spectra of the prepared OS; however, their relative intensities were so low that it was not possible to assign them to any particular structure.

The aldehyde content per unit mass of OS was then determined by titration with hydroxylamine hydrochloride [21], giving a value of 11.4 mmol∙g^−1^. Considering that the prepared OS is a mixture of two main products (i.e., 42 and 58 mol%), the aldehyde content should be 12.3 mmol∙g^−1^, thereby indicating that the purity of the resulting OS was 92%.

In previous reports, the aldehyde content of OS was determined to be ~20–45% for full oxidation [19,20,21,22,23,24,25]. In contrast, the aldehyde purity obtained in this study (92%) was significantly higher. This improvement was likely due to product purification by anion and cation exchange chromatography after sucrose oxidation, which removed any ionic byproducts (e.g., formic acid) to dramatically improve the purity of the product. 

As shown in Figure 2, the integral value for the C5 resonance of the fully oxidatively cleaved glucose residue of OS(II) at 74 ppm and that of the C6 resonance of the partially oxidatively cleaved glucose residue of OS(I) at 60 ppm, which do not overlap with other resonances, were 0.42 and 0.58, indicating that the compositions of OS(I) and OS(II) were 52 and 48 mol%, respectively. In addition, the integral value of 3.96 obtained for the aldehyde carbon atoms confirmed that both OS(I) and OS(II) possess four aldehyde groups in their structures.

### 2.2. Preparation of CMC Hydrogels Using OS

**Figure 4:**
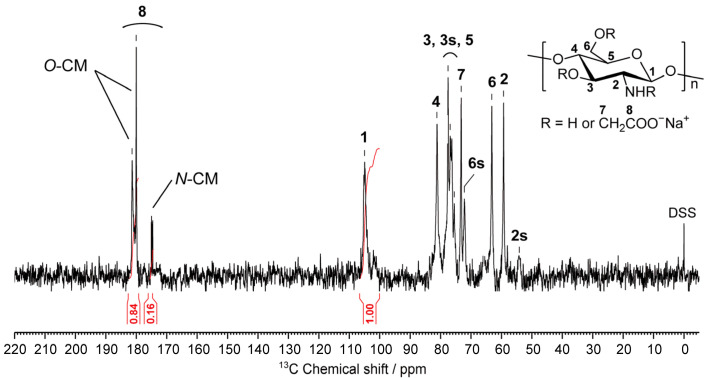
Quantitative ^13^C NMR spectrum of CMC used for preparation of CMCG (298 K, D_2_O). Resonance assignments are also indicated. The numbers 2s, 3s, and 6s represent the resonance lines at the positions substituted by the carboxymethyl groups. *O*-CM and *N*-CM refer to the carbonyl carbon resonances of the carboxymethyl groups substituted at the hydroxyl and amine groups of chitosan, respectively.

Prior to the preparation of the CMCG, the structure of CMC was characterized. Thus, Figure 4 shows the quantitative ^13^C NMR spectrum of the CMC dissolved in D_2_O. Each resonance line of the CMC was assigned, as indicated in the figure. In addition, no signal corresponding to the methyl groups of the chitosan GlcNAc residues was observed (22 ppm) [11], indicating that almost all the constituent residues of CMC consist of GlcN residues. Furthermore, the carbonyl carbon signals of the carboxymethyl groups substituted at the amino and hydroxyl groups of chitosan were observed at 180 and 175 ppm [31] with integral values of 0.84 and 0.16, respectively (c.f., an integral of 1 for the anomeric carbon signal at 108–101 ppm). These integral values represent the degree of substitution (DS) at the hydroxyl and carboxymethyl groups of CMC, and so the total DS was determined to be 1.0. Therefore, based on the NMR data, the average molecular weight and the average number of free amino groups per GlcN residue of CMC were determined to be 241 g mol^−1^ and 0.84, respectively. 

Following its structural determination, CMC was used to prepare CMCG by reaction with OS. A series of CMCG samples (CMCG 1–4) was prepared from the dissolution of CMC and OS in deionized water in the absence of a catalyst. Table 2 summarizes the feed amounts of CMC and OS employed for the preparation of CMCG 1–4, where the concentration of CMC was 2 *w*/*v*% and the molar aldehyde contents of OS were 0.5-, 1-, 2-, and 4-times those of the free amino groups of CMC, respectively.

**Figure 5:**
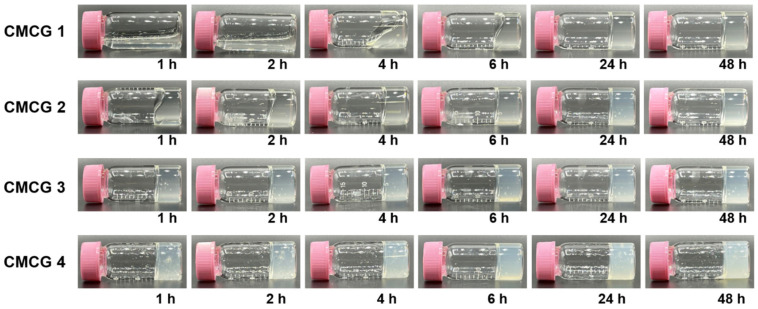
Photographic images of the reaction mixtures for the preparation of carboxymethyl chitosan hydrogels (CMCG 1–4) after 1, 2, 4, 6, 24, and 48 h of cross-linking between carboxymethyl chitosan and the oxidized sucrose.

Figure 5 shows photographic images of the reaction mixtures during the preparation of CMCG 1–4 after 1, 2, 4, 6, 24, and 48 h of reaction. As indicated, all mixtures began to gel immediately after the reaction; CMCG 1 and 2 completely gelatinized within 24 and 4 h, respectively, while CMCG 3 and 4 lost their fluidity within 1 h. These results indicate that OS functions as a cross-linking agent for CMC without the requirement for a catalyst. After 48 h of reaction, the obtained hydrogels were purified by washing them with water to remove any unreacted OS. Finally, they were lyophilized to obtain CMCG 1–4 as white solids.

### 2.3. Structural Characterization of the Prepared CMCG Specimens

**Figure 6:**
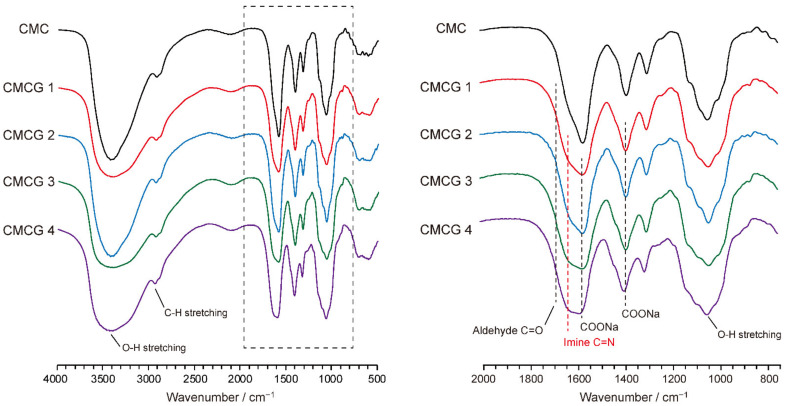
Full and expanded Fourier transform infrared spectra of CMCG 1–4 and CMC.

Figure 6 shows the FTIR spectra of CMCG 1–4, along with that of CMC for reference. As can be seen, the spectrum of CMC exhibits absorption peaks corresponding to the asymmetric and symmetric stretching vibrations of the carboxylate groups at 1595 and 1401 cm^−1^, respectively [34]. In the spectra of CMCG 1–4, in addition to these two absorption signals for the carboxylate groups, a new absorption peak attributed to the C=N stretching vibration of the imine group was observed at 1680–1650 cm^−1^ [28]. Furthermore, the C=O stretching peak of the aldehyde groups detected at 1710 cm^−1^ in the FTIR spectrum of OS (Figure S1) was not detected in the CMCG samples, suggesting that the aldehyde groups of OS reacted with the amine groups of CMC to form imine bonds.

**Figure 7:**
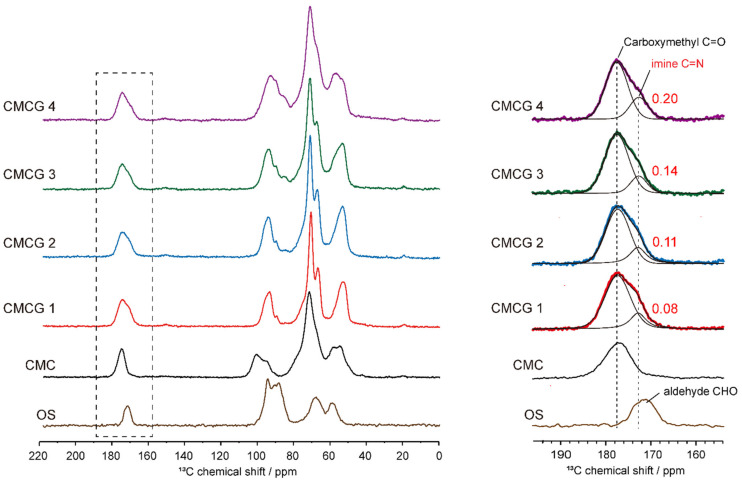
Quantitative solid-state ^13^C NMR spectra of CMCG 1–4, CMC, and OS (left-hand spectra), and the results of lineshape analysis for the carbonyl carbon region (right-hand spectra).

For a quantitative discussion of imine formation in the CMCG samples, quantitative solid-state ^13^C NMR spectra were recorded for the CMCG 1–4 specimens and for the starting materials (i.e., CMC and OS) (Figure 7). Focusing on the carbonyl carbon region at 190–160 ppm, OS aldehyde groups were detected at 171 ppm, while the CMC carboxylate groups were observed at 178 ppm. In contrast, in the spectra of the CMCG samples, the resonance of the imine was detected at 173 ppm as a shoulder of the carboxylate line at 178 ppm. Lineshape analysis with Lorentzian lines [35,36] was then applied to the partially overlapping imine and carboxylate resonances of CMCG 1–4, as presented in Figure 7. The integral values of the imine resonances for CMCG 1–4 were 0.08, 0.11, 0.14, and 0.20, respectively (carboxylate resonance = 1). Since the DS of carboxymethyl groups per GlcN unit in CMC is 1.0, the integral values of the imine carbon resonances indicate the number of imine bonds formed per GlcN unit. Defining these values as the cross-linking degree (CR), the CR values for CMCG 1–4 were determined to be 0.08, 0.11, 0.14, and 0.20, respectively, thereby indicating that an increase in the feed amount of OS enhances the CR of the CMCG specimens.

### 2.4. Thermal Properties of CMCG

**Figure 8:**
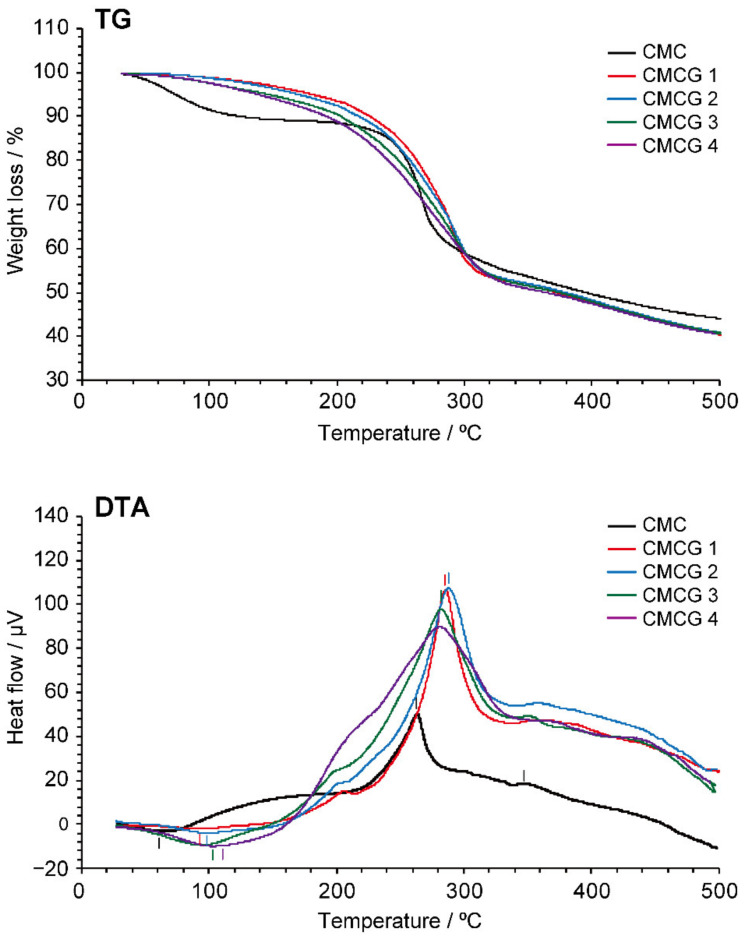
Thermogravimetric (TG) and differential thermal analysis (DTA) traces for CMCG 1–4 and CMC.

Figure 8 shows the thermogravimetry (TG)/differential thermal analysis (DTA) traces of CMCG 1–4 and CMC. More specifically, the TG/DTA trace of CMC shows that thermal decomposition occurs through three main weight-loss processes: the first is due to vaporization of the absorbed and intermolecularly hydrogen-bonded water (60–160 °C), the second mainly corresponds to the dehydration and decomposition of the functional groups and glycosidic linkages of CMC through pyrolysis (200–280 °C), and the third is the degradation of the skeleton of the CMC monomer (>280 °C) [37]. This thermal behavior was confirmed by the observation of an endothermic peak at 62 °C and exothermic peaks at 259 and 350 °C in the DTA trace. In the case of CMCG, although the mass loss due to water evaporation in the first stage could not be clearly distinguished from the polymer degradation in the second stage, endothermic peaks were detected for CMCG 1–4 at 96, 99, 103, and 113 °C, respectively. This finding indicates that the affinity between CMCG and the absorbed and intermolecular hydrogen-bonded water was enhanced with an increase in the CR of CMCG. In addition, the exothermic peak at 259 °C, which corresponds to the second degradation stage of CMC, shifted to 280–300 °C in the DTA traces of the CMCG samples, suggesting that the formation of cross-linking between the CMC chains improved the thermal stability. Moreover, degradation of the CMCG monomer proceeded between 320 and 500 °C, similar to in the case of CMC degradation, although no clear endothermic peaks were observed.

**Figure 9:**
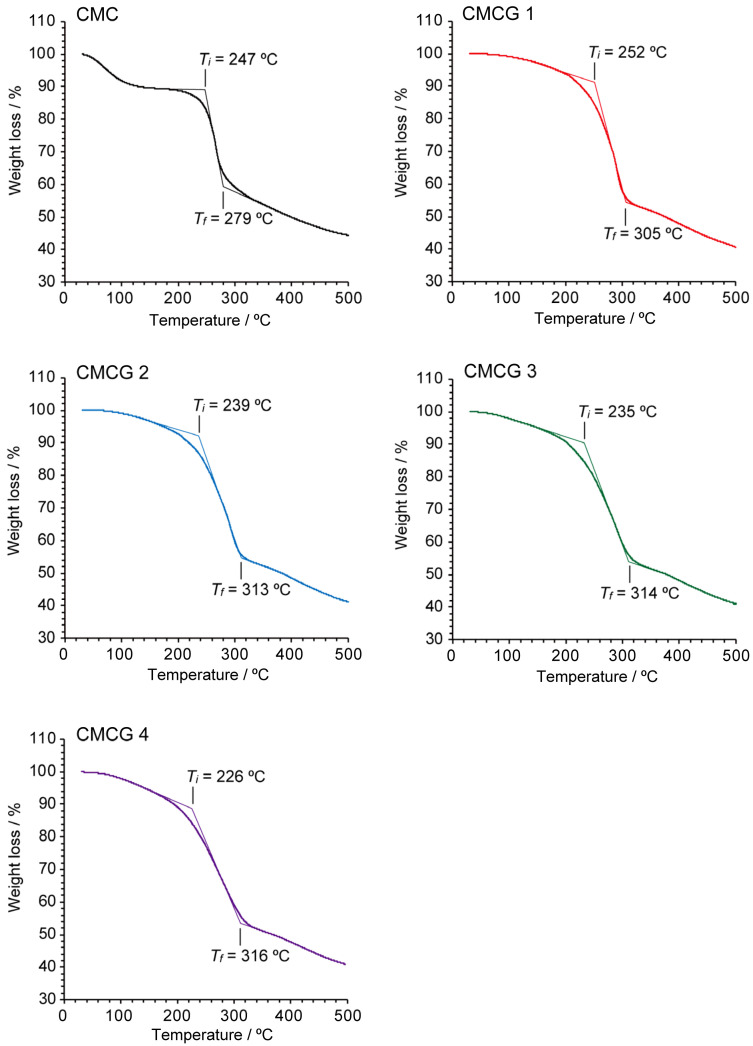
The initial (*T_i_*) and final (*T_f_*) degradation temperatures for the second stage of thermal decomposition for CMCG 1–4 and CMC determined by thermogravimetric analysis (Figure 8, top).

The most remarkable difference between the thermal behaviors of CMCG and CMC was observed during the second stage of thermal decomposition. The initial (*T*_i_) and final (*T*_f_) decomposition temperatures for the second degradation of the samples are shown in Figure 9. More specifically, the *T*_i_ and *T*_f_ of CMC were determined to be 247 and 279 °C, respectively, whereas the CMCG 1–4 specimens exhibited a decreasing trend for *T*_i_ and an increasing trend for *T*_f_ with an increase in the CR. These tendencies were previously reported for other chemically cross-linked polysaccharide hydrogels [38], wherein chemical cross-linking between the polysaccharide chains was found to enhance the molecular structure of the hydrogel, thereby resulting in an increased value of *T*_f_. On the other hand, introducing cross-linking decreases the number of interactions (e.g., hydrogen bonds) between the polysaccharide chains, lowering the hydrogel density and reducing the *T*_i_ value. Therefore, the cross-linking imparted by OS appeared to increase the structural stability of CMCG while reducing the number of interactions between the CMC molecular chains. Moreover, while the CMC lost 53% of its weight at 500 °C, the CMCG 1–4 specimens exhibited a mass loss of 58% at the same temperature. The slight difference suggests further confirms the presence of reduced interactions between CMC chains due to the introduction of cross-linking by OS.

### 2.5. Water Absorbency of the CMCG Specimens

**Figure 10:**
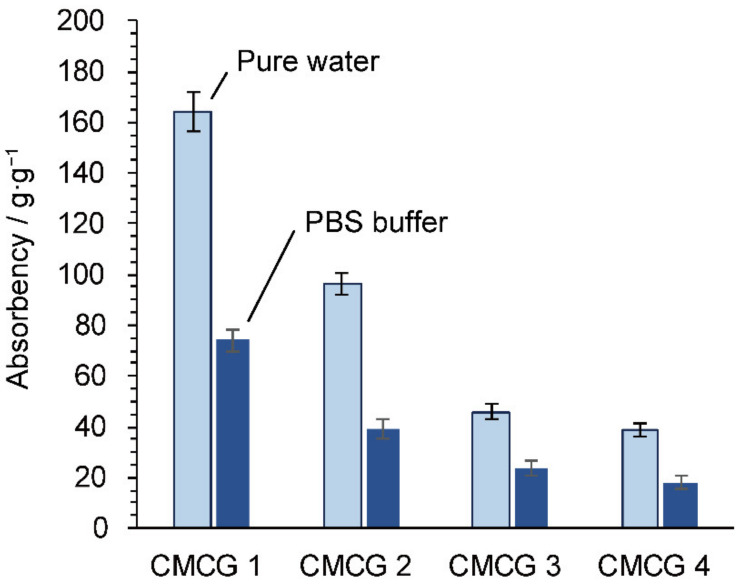
Absorbency of the CMCG 1–4 species toward pure water and phosphate-buffered saline after 24 h at 298 K.

In general, the absorption of water by ionic gels is affected by the osmotic pressure created by the difference in the mobile cation concentration between the gel and the absorbing solution [34]. As shown in Figure 10, the absorbency of the prepared CMCG hydrogels in phosphate-buffered saline (1×, pH 7.4) was significantly lower than that in pure water. Thus, the CMCG sample exhibited the typical behavior of an ionic gel, and its water absorbency was attributed to the osmotic pressure generated by free sodium cations and the electrostatic repulsion between CMC molecular chains owing to the presence of carboxylic acid anions [39]. In addition, the absorbency decreased as the CR of CMCG increased; this increase in the CR restricted the mobility of the CMC chains in the hydrogels, and the CMC chains became closer to one another, thereby creating fewer voids and contributing to reduced water absorption.

### 2.6. Scanning Electron Microscopy (SEM) Observations of CMCG

**Figure 11:**
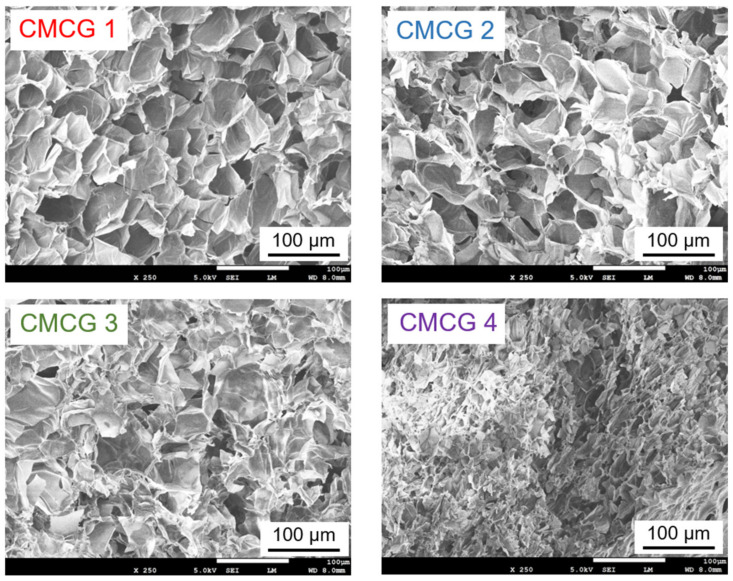
Scanning electron microscopy (SEM) images for the cross-sectional surface of the CMCG 1–4 species swelled in PBS.

Figure 11 shows the scanning electron microscopy (SEM) images of the cross-sections of the lyophilized CMCG samples after saturation absorption in PBS. These images demonstrate that the hydrogels exhibited a cellular structure composed of macropores, wherein the sizes of these macropores depended on the sample CR. More specifically, a higher CR led to a decreased macropore size, which is consistent with the result that an increase in the CR resulted in a decrease in the water absorbency.

### 2.7. Rheological Properties of the CMCG Specimens

**Figure 12:**
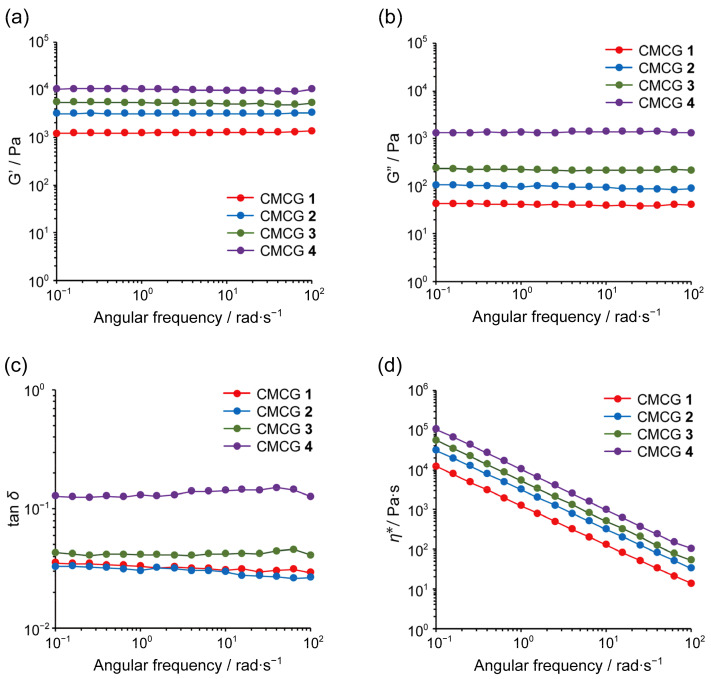
Rheological properties of CMCG at 298 K following swelling and saturation with PBS. The (**a**) storage modulus (G′), (**b**) loss modulus (G″), (**c**) tan *δ* (= G″/G′), and (**d**) complex viscosity (ƞ*) profiles.

Figure 12 shows the plots of the storage (G′) and loss (G″) moduli, the tan *δ* (=G″/G′) values, and the complex viscosities (ƞ*) against the angular frequency sweeps for the CMCG 1–4 specimens following PBS saturation. For each hydrogel, G′ was always higher than G″, and no cross-over points (tan *δ* = 1) were detected between G′ and G″ for any of the CMCG samples. In addition, the tan δ values for these hydrogels were in the range of 0.03–0.2, indicating that the elastic properties were superior to the viscous properties in the dynamic viscoelastic behavior of the CMCG specimens [34]. These observations indicate that the CMCG samples were typical chemical gels with the characteristics of a permanent gel network. Furthermore, the rheological measurements showed that an increase in the CR resulted in higher G′ and G″ values, ultimately leading to an increase in ƞ*. It was therefore apparent that the viscoelastic behaviors of CMCG strongly depend on the CR of the hydrogel, and that the inclusion of a large amount of OS caused a significant increase in the G′ and G″ moduli and in ƞ*.

**Figure 13:**
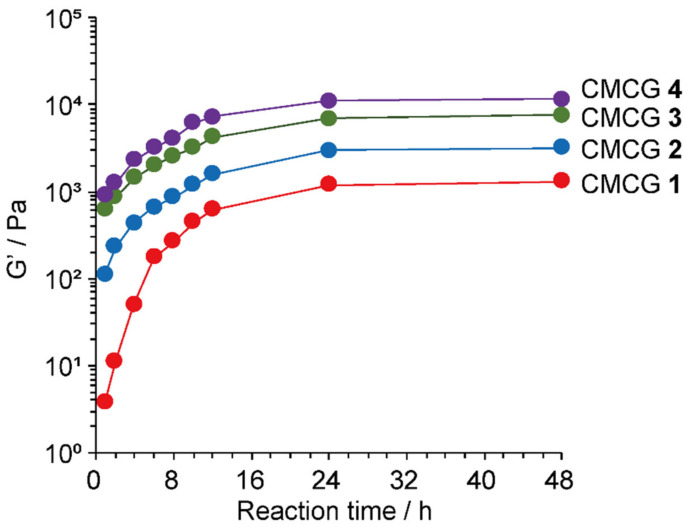
Storage moduli (G′) of CMCG 1–4 at 298 K obtained by varying the cross-linking reaction time. The G′ values were recorded at an angular frequency of 1 rad∙s^−1^. All CMCG samples had previously undergone swelling and saturation with PBS.

Finally, the G′ at an angular frequency of 1 rad∙s^−1^ was plotted against the reaction time for each hydrogel (Figure 13). As shown in the figure, the elastic properties of the CMCG specimens increased rapidly after the start of the cross-linking reaction and remained constant after 24 h, thereby indicating that the cross-linking reaction essentially reached completion within 24 h, and suggesting that a further extension of the reaction time should not significantly affect the gel properties.

## 3. Materials and Methods

### 3.1. Materials

Sucrose, sodium periodate, barium chloride dihydrate, hydroxylamine hydrochloride, 0.1 *w*/*v*% methyl orange solution, and PBS (1×, pH7.4) were of a chemically pure grade and were purchased from FUJIFILM Wako Pure Chemical Co. (Osaka, Japan). Fine powdered CMC (deacetylation degree, 99.9%) was purchased from Santa Cruz Biotechnology, Inc. (Dallas, TX, USA). All other chemicals and solvents were of chemically pure grade and were used as received.

### 3.2. Preparation and Structural Characterization of OS

#### 3.2.1. Preparation

Sucrose (6.0 g, 17.5 mmol) and sodium periodate (11.3 g, 52.5 mmol) were dissolved in deionized water (200 mL), and the mixture was stirred at 298 K for 26 h. After this time, barium dichloride (6.42 g, 26.2 mmol) was added, and the mixture was stirred at 278 K for 1 h to allow complete precipitation of the barium iodate. The resulting mixture was then filtered to obtain the supernatant solution containing the prepared OS. The supernatant solution was passed through a cation exchange column filled with an anion exchange resin (Amberlite FPC3500, Organo Co., Tokyo, Japan) adjusted to the OH type, and then passed through a cation exchange column filled with a cation exchange resin (Amberlite IRA402BLCl, Organo Co.) to remove formic acid, any remaining barium chloride, and the periodate ions. The resulting solution was freeze-dried to obtain the prepared OS as a white powder.

#### 3.2.2. Aldehyde Quantification Using the Titration Method

The aldehyde content of the prepared OS was estimated using a previously described titration method [21]. More specifically, OS (0.1 g) was dissolved in a 0.25 mol L^−1^ hydroxylamine hydrochloride solution (25 mL), one drop of methyl orange indicator was added to the solution, and the solution was stirred at 298 K for 2 h. After this time, the solution was titrated against a 0.1 mol L^−1^ aqueous solution of sodium hydroxide. At the endpoint, the color of the solution changed from red to yellow, and the number of moles of sodium hydroxide that had reacted was determined to represent the number of moles of aldehyde groups present in the sample. The percentage degree of oxidation was calculated from the number of moles of aldehyde groups. All experiments were performed in triplicate.

#### 3.2.3. Structural Characterization

The FTIR spectra were recorded on a Spectrum II FTIR spectrometer (PerkinElmer, Inc., Waltham, MA, USA) at a resolution of 1 cm^−1^ in the 4000–500 cm^−1^ range, and averaged over 16 scans. The solution-state NMR spectra were recorded at 298 K on a Bruker AVIII spectrometer (^1^H frequency of 500.13 MHz, ^13^C frequency of 125.13 MHz, Karlsruhe, Germany) equipped with a 2-channel 5 mm BBFO probe incorporating a *z*-gradient coil. Each sample (20–40 mg) dissolved in D_2_O (800 μL, 99.9% isotopic purity, Sigma-Aldrich Inc., St. Louis, MO, USA) in a 5 mm glass NMR tube (Wilmad-Labglass Co., Vineland, NJ, USA). The one-dimensional ^1^H, ^13^C, ^13^C DEPT135, and quantitative ^13^C NMR spectra were recorded using Bruker BioSpin default pulse programs. For the quantitative ^13^C NMR experiment [31], the excitation pulse for the ^13^C nuclei with a flip angle of 30°, the data acquisition time, the repetition time, and the number of scans were set to 5 μs, 2.71 s, 30 s, 10, 240, respectively. The two-dimensional ^1^H–^13^C HSQC spectra were recorded for a dataset of 2048 (*t*_2_) × 256 (*t*_1_) by conducting 8 scans per *t*_1_ increment. Two-dimensional ^1^H–^13^C HSQC−TOCSY data were obtained for a dataset of 2048 (*t*_2_) × 256 (*t*_1_) by performing 16 scans per *t*_1_ increment. In the HSQC–TOCSY experiments, the interpulse delay of the HSQC sequence, the Hartman–Hahn spin-locking time, and the repetition time were set to 3.6 ms, 100, and 1.5 s, respectively. The ^1^H and ^13^C chemical shifts of the obtained NMR spectra were calibrated by assigning the methyl peak of the DSS internal standard to 0 ppm.

### 3.3. Preparation and Characterization of the CMCG Specimens

#### 3.3.1. Preparation

To prepare a series of CMCG samples, CMC (400 mg, 1.75 mmol for an averaged monomer unit) was dissolved in deionized water (10 mL). Subsequently, the prepared OS (6.10 mg) was dissolved in deionized water (10 mL) and this solution was added dropwise to the CMC solution with stirring at 298 K for 10 min. After allowing the resulting solution to stand at 298 K for 24 h, the reaction mixture was subjected to dialysis against a deionized water stream (dialysis membrane tube, Mw 12,000 cut-off, Thermo Fisher Scientific, USA) for 3 d. The obtained hydrogel was freeze-dried to obtain the desired CMCG 1. The above procedure was repeated to prepare CMCG 2–4, wherein the feed amount of OS was changed to 12.2, 24.5, and 49.0 mg, respectively.

#### 3.3.2. Structural Characterization

The FTIR spectra of the CMCG samples were recorded using a similar method to that described for the OS. The solid-state ^13^C NMR spectra were recorded at 298 K on a Bruker AVIII spectrometer using a 4 mm dual-tuned MAS probe (^1^H frequency of 500.13 MHz, ^13^C frequency of 125.13 MHz, Karlsruhe, Germany) at a MAS frequency of 10 kHz. For quantitative analysis, the solid-state NMR spectra were acquired using the dipolar-decoupled/magic angle spinning method [40]. The ^13^C-excitation pulse length (flip angle of 30°), the data acquisition time, and the repetition time were set to 1.5 μs, 20 ms, and 30 s, respectively. During the data acquisition period, a small phase incremental alternation with 64 proton decoupling steps was applied with a ^1^H field strength of 100 kHz. Spectra were collected using 2048 scans. The chemical shifts of the ^13^C spectra were calibrated based on the carbonyl carbon resonance of D-glycine (176.03 ppm), which was used as an external reference. Lineshape analyses of the carbonyl carbon resonances were performed using a nonlinear least-squares method [35,40] to fit the ^13^C resonance lines with a Lorentzian function. For this purpose, the Sola simulation software was employed (TopSpin software package, v. 3.8, Bruker BioSpin). Each fit was performed using an initial Lorentzian line with a full width at half maximum of 400 Hz. The line amplitude was manually adjusted to match the experimental spectrum and achieve a coefficient of determination *r*^2^ greater than 0.88. Subsequently, the full width at half-maximum values and amplitudes of the Lorentzian lines were fitted using a simplex algorithm [35] to minimize the least-squares differences between the experimental and simulated spectra. The quality of fit was set at *r^2^* > 0.96.

#### 3.3.3. TG/DTA

The TG/DTA profiles were acquired on an EVO2 TG 8120 Plus thermogravimetric dynamic thermal analyzer (Rigaku Co., Akishima, Japan) using an Al_2_O_3_ crucible under a nitrogen gas flow. Each CMCG sample weighed 7 mg and the thermograms were obtained in the 50–500 °C range at a heating rate of 5 °C∙min^−1^.

#### 3.3.4. Water-Absorbency Tests

The water absorbencies of the CMCG specimens were measured via gravimetric analysis. Each CMCG sample of a fixed weight was immersed in a large amount of deionized water or PBS at 298 K for 24 h. The swollen sample was then filtered through a 255-mesh nylon screen to separate it from any unabsorbed water. The equilibrium water absorbency of the CMCG was determined using the following equation:

(water absorbency/g∙g^−1^) = (*m*_2_ − *m*_1_)/*m*_1_,

where *m*_1_ and *m*_2_ are the weights of the samples in the dry and swollen states, respectively. All experiments were performed in triplicate and the results were averaged.

#### 3.3.5. SEM Observations

SEM images of the cross-sectional surfaces of the swollen CMCG specimens were obtained following sample preparation as follows. After swelling in PBS at 298 K for 24 h, each CMCG was carefully cut using a razor blade, frozen at −70 °C, and freeze-dried under vacuum. The freeze-dried samples were fixed on specimen stubs that had been sputter-coated with a layer of platinum (4 nm thickness) prior to observation. The SEM images were obtained using a JEOL JSM-7500F scanning electron microscope (JEOL Ltd., Akishima, Japan) at an acceleration voltage of 5.0 kV.

#### 3.3.6. Rheological Measurements

The rheological behaviors of the CMCG specimens swelled in PBS at 298 K for 24 h were evaluated in triplicate using a Physica MCR 301 rheometer (Anton Paar GmbH, Graz, Austria) equipped with a 25 mm parallel-plate measuring geometry, a Peltier device for temperature control, and a Rheoplus 32 data analyzer. The gap and stain were set to 1.0 mm and 1.0%, respectively. The oscillatory shear responses at 298 K were determined at 0.1 Pa over the frequency range of steady shear tests with an angular frequency range of 10^−1^–10^2^ rad∙s^−1^.

## 4. Conclusions

To elucidate the structure of oxidized sucrose (OS), two-dimensional nuclear magnetic resonance (NMR) spectroscopy was employed, and the molecular structures of the two main products of OS, namely, OS(I) and OS(II), were experimentally revealed for the first time. More specifically, complete chemical shift analysis indicated that both OS(I) and OS(II) exhibited oxidative cleavage of the C3–C4 bond of fructose, while OS(I) exhibited oxidative cleavage of the C2–C3 bond of glucose, although, in OS(II), the C2–C3–C4 bonds were fully oxidized. It was also deduced that both OS(I) and OS(II) contain four aldehyde groups, and their compositions were successfully quantified by NMR experiments. Furthermore, we investigated the cross-linking of OS to carboxymethyl chitosan (CMC) in the absence of a catalyst to produce a series of CMC hydrogels (CMCG 1–4). Solid-state NMR spectroscopic characterization of these CMCGs revealed that the aldehyde group of OS selectively cross-linked with the amino group of CMC to form an imine. In addition, the degree of cross-linking of the hydrated gels increased with an increase in the amount of OS added during CMCG synthesis. As a result, the degree of water absorption and the dynamic modulus of the gel increased. From our evaluation of the dynamic viscoelasticity of the prepared gels, it was confirmed that the cross-linking reaction began immediately, and reached completion within 24 h. Thus, these results demonstrate that OS can be employed as a natural cross-linking agent for the selective cross-linking of amino sugars, including β-(1→4)-glucosamine residues, such as hyaluronic acid and heparin, and galactosamine residues, such as chondroitin sulfate and dermatan sulfate. We therefore expect that the use of OS will facilitate the synthesis of polysaccharide gels. Since these polysaccharides play various roles in the body, including acting as an extracellular matrix and a scaffold for cell adhesion, whilst also retaining and providing cell growth factors, it is likely that mucopolysaccharide gels cross-linked by OS could be prepared for medical applications. Furthermore, if biological safety can be ensured, such systems have the potential for use as drug delivery carriers.

## Figures and Tables

**Table 1:** ^1^H and ^13^C chemical shifts of the prepared OS (298 K, D_2_O).

OS	Residue ^1^	H1/C1	H2/C2	H3/C3	H4/C4	H5/C5	H6/C6
OS(I)	Glc	4.91/90.7	8.41/173.1	8.41/173.1	4.05/72.9	4.98/91.5	4.01, 3.71/60.3
	Fru	3.94/63.5	n.d./97.6	8.41/173.1	8.41/173.1	5.06/93.8	3.80/62.2
OS(II)	Glc	5.16/88.5	8.41/173.1		8.41/173.1	3.73/74.2	3.79/62.2
	Fru	3.94/63.5	n.d./97.6	8.41/173.1	8.41/173.1	5.06/93.8	3.80/62.2

^1^ Oxidatively cleaved glucose (Glc) and fructose (Fru) units of sucrose. n.d = not detected.

**Table 2:** Initial feed amounts of carboxymethyl chitosan (CMC) and oxidized sucrose (OS) for preparation of the CMG hydrogels (CMCG 1–4). The cross-linking degrees (CR) of the hydrogels are also given.

Sample	CMC/mg (Amine/mmol)	OS/mg (Aldehyde/mmol)	CR ^1^
CMCG 1	400 (0.14)	6.10 (0.07)	0.08
CMCG 2	400 (0.14)	12.2 (0.14)	0.11
CMCG 3	400 (0.14)	24.5 (0.28)	0.14
CMCG 4	400 (0.14)	49.0 (0.56)	0.20

^1^ Number of imine bonds formed between CMC and OS per GlcN residue.

## Data Availability

The data presented in this study supporting the results are available in the main text. Additional data are available upon reasonable request from the corresponding author.

## Supplementary Materials

The following supporting information can be downloaded at: https://www.mdpi.com/article/10.3390/molecules27186137/s1, Figure S1: Fourier transform infrared spectra of the oxidized sucrose (OS) and sucrose; Figure S2: ^13^C and ^13^C distortionless enhancement by polarization transfer 135 (DEPT-135) nuclear magnetic resonance (NMR) spectra of the oxidized sucrose (OS) in the range of 110–55 ppm.
