# Supplementary material for: Detailed Structural Characterization of Oxidized Sucrose and Its Application in the Fully Carbohydrate-Based Preparation of a Hydrogel from Carboxymethyl Chitosan
Source: Molecules. 2022 Sep 19;27(18):6137. doi: 10.3390/molecules27186137 (PMC9503324; doi:10.3390/molecules27186137)
Supplement: Supplementary file 1 [file molecules-27-06137-s001.zip › molecules-1909654-supplementary.pdf]

molecules

Supplementary Materials

Detailed Structural Characterization of Oxidized Sucrose and Its Application in the Fully Carbohydrate-Based Preparation of a Hydrogel from Carboxymethyl Chitosan

Hiroyuki Kono [1,*], Junki Noda [1], Haruki Wakamori [2]

[1] Division of Applied Chemistry and Biochemistry, National Institute of Technology, Tomakomai College, Nishikioka 443, Tomakomai 059 1275, Japan
[2] Hokkaido Soda Co. Ltd., Numanohata 134-122, Tomakomai 059 1364, Japan
* Correspondence: kono@tomakomai-ct.ac.jp; Tel.: +81-144-67-8036

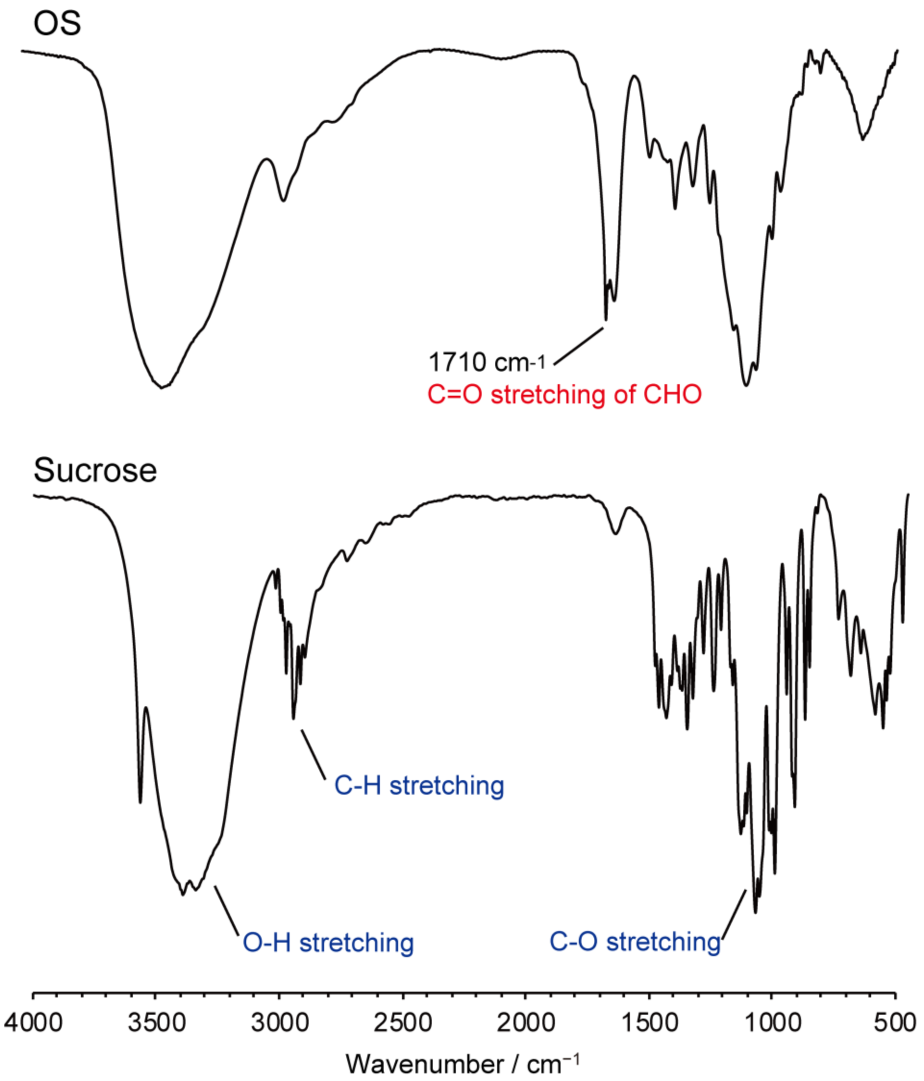

Figure S1. Fourier transform infrared spectra of sucrose and the oxidized sucrose (OS).

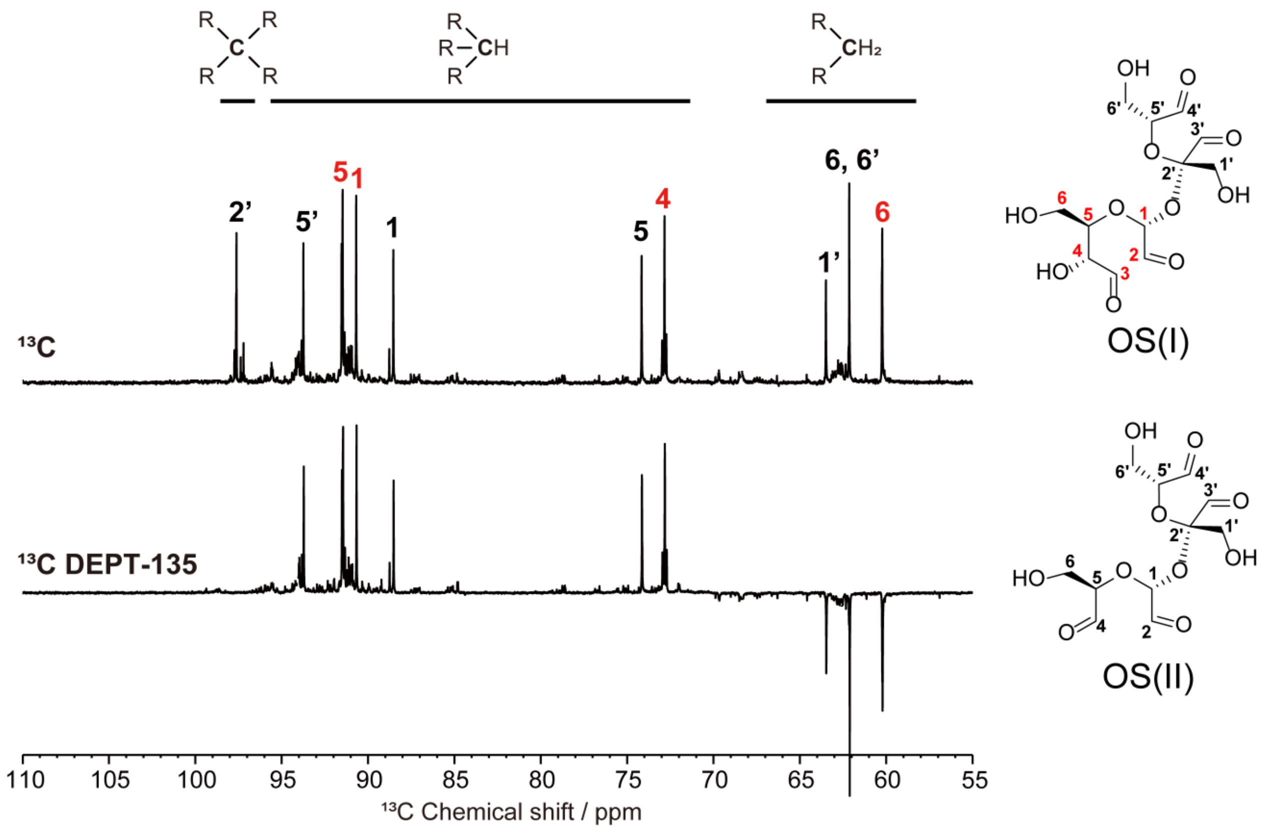

Figure S2. ^{13}C and ^{13}C distortionless enhancement by polarization transfer 135 (DEPT-135) nuclear magnetic resonance (NMR) spectra of oxidized sucrose (OS) in the range of 55–110 ppm with assignment of the two main products, OS(I) and OS (II), as indicated in the spectra.
